# Induction of miR-96 by Dietary Saturated Fatty Acids Exacerbates Hepatic Insulin Resistance through the Suppression of INSR and IRS-1

**DOI:** 10.1371/journal.pone.0169039

**Published:** 2016-12-30

**Authors:** Won-Mo Yang, Kyung-Ho Min, Wan Lee

**Affiliations:** 1 Department of Biochemistry, Dongguk University College of Medicine, Gyeongju, Korea; 2 Endocrine Channelopathy, Channelopathy Research Center, Dongguk University College of Medicine, Goyang, Korea; Brown University Warren Alpert Medical School, UNITED STATES

## Abstract

Obesity is defined as the excessive accumulation of body fat that ultimately leads to chronic metabolic diseases. Diets rich in saturated fatty acids (SFA) exacerbate obesity and hepatic steatosis, which increase the risk of hepatic insulin resistance and type 2 diabetes (T2DM). Although microRNAs (miRNAs) play an important role in a range of biological processes, the implications of SFA-induced miRNAs in metabolic dysregulation, particularly in the pathogenesis of hepatic insulin resistance, are not well understood. This study investigated the implications of miR-96, which is induced strongly by SFA, in the development of hepatic insulin resistance. The liver of HFD mice and the palmitate-treated hepatocytes exhibited an impairment of insulin signaling due to the significant decrease in INSR and IRS-1 expression. According to expression profiling and *q*RT-PCR analysis of the miRNAs, the expression level of miR-96 was higher in hepatocytes treated with palmitate. Moreover, miR-96 was also upregulated in the liver of HFD mice. Interestingly, miR-96 targeted the 3’UTRs of *INSR* and *IRS-1* directly, and repressed the expression of INSR and IRS-1 at the post-transcriptional level. Accordingly, the overexpression of miR-96 was found to cause a significant decrease in INSR and IRS-1 expression, thereby leading to an impairment of insulin signaling and glycogen synthesis in hepatocytes. These results reveal a novel mechanism whereby miR-96 promotes the pathogenesis of hepatic insulin resistance resulted from SFA or obesity.

## Introduction

Obesity is a rapidly spreading chronic health problem resulting from an imbalance between energy intake and energy output, which often leads to a range of metabolic diseases [1, 2]. The excess intake of dietary saturated fatty acids (SFA), which is the leading cause of weight gain and obesity, inevitably increases intracellular lipid accumulation in the liver and skeletal muscle [2, 3]. Because the liver is considered to be the most important organ for metabolic fuel homeostasis, the buildup of lipid droplets within the liver can cause metabolic dysregulation to varying degrees as well as a progressive complex of liver disease, known as nonalcoholic fatty liver disease (NAFLD) [4]. NAFLD is now the most prevalent liver disorder in the developed countries and is associated strongly with the development of hepatic insulin resistance and reduced whole-body insulin sensitivity. The hepatic insulin resistance derived from NAFLD generally implies the insufficient ability of insulin to suppress glycogenolysis, gluconeogenesis, and glucose output in the liver, thereby causing decreases in glucose disposal, consequently leading to type 2 diabetes (T2DM), and metabolic syndrome [2, 3]. Accumulating studies conducted on obese humans and rodent models have suggested a range of causal associations between NAFLD and insulin resistance in the liver and other tissues [2–4]. Regardless of progress, the exact mechanism for how SFA provokes hepatic insulin resistance is not well understood.

Insulin signaling includes a finely regulated relay of intracellular signals, that mostly involves the phosphorylation and dephosphorylation of signaling molecules, which are initiated from insulin binding to the insulin receptor (INSR) [3, 5]. The binding of insulin to the INSR induces tyrosine phosphorylation of the insulin receptor substrate (IRS), and then transduces signals through the downstream enzymes, such as PI3K and Akt2 [6]. Thus far, several causes have been proposed to explain how the dysregulation of insulin signaling processes arises in NAFLD in a variety of experimental and clinical models [5, 7]. The accumulation of SFA increases intracellular lipid metabolites including ceramide and DAG, which impair the insulin signaling cascade through the IRS-1 serine phosphorylation induced by PKC, IKK and JNK [2, 8]. Although IRS-1 serine phosphorylation by SFA is considered as an emerging detrimental factor in insulin sensitivity, a growing lines of evidence have suggested that the reduction of INSR expression also promotes the pathogenesis of insulin resistance and diabetes. The knockout model of INSR in mice exhibited the rapid onset of hyperinsulinemia and hyperglycemia, followed by diabetic ketoacidosis [9], and the liver-specific INSR knockout in mice also showed severe liver dysfunction, hyperglycemia, hyperinsulinemia, and impaired glucose homeostasis [7]. Furthermore, an accumulating evidence has been reported a modest suppression of INSR expression in T2DM patients [10, 11]. Therefore, the level of hepatic INSR expression is strongly associated with the whole-body insulin sensitivity. On the other hand, the molecular mechanisms responsible for obesity or SFA-induced downregulation of INSR are largely unknown, even though different hypotheses have been put forward.

MicroRNAs (miRNAs) are small non-coding RNAs that regulate gene expression at the post-transcriptional level [12]. Mature miRNAs bind to specific sequences located on the 3’ untranslated regions (3’UTR) of the target genes, eventually triggering the suppression of translation or degradation of the target mRNAs [12, 13]. Although the molecular targets and roles of the individual miRNAs are still largely unknown, it has been suggested that the dysregulation of miRNAs expression is closely associated with a range of pathological states, such as neurodegeneration and cardiovascular diseases and cancer [13, 14]. Following the discovery that miRNA plays an important role in metabolic regulation, such as amino acids catabolism [15], miRNAs have been also suggested to be critical regulators in the glucose and lipid metabolism, whose derangement is linked to the development of insulin resistance and T2DM [16, 17]. Recently, it was reported that the certain miRNAs targeting the 3’UTR of the insulin signaling intermediates’ mRNA are modulated by the SFA-induced obesity and NAFLD, and that these miRNAs participate actively in the development of hepatic insulin resistance [18–20]. Therefore, it is noteworthy that high fat diet (HFD) in mice causes insulin resistance, concomitant with the upregulation of specific miRNAs, such as miR-802, miR-103, and miR-107, in the liver [18, 21]. Despite the advances in knowledge, the emerging mechanism for how miRNAs are linked causally in the hepatic insulin resistance by SFA-induced obesity is poorly understood. In this study, a significant increase in miR-96 expression was found in the liver of HFD mice and SFA-treated hepatocytes, suggesting that miR-96 is involved in the pathogenesis of hepatic insulin resistance. Therefore, we further revealed that miR-96 targets 3’UTRs of *INSR* and *IRS-1* genes directly to suppress the expression of the INSR and IRS-1 protein, resulting in impaired insulin signaling and glycogen synthesis. Overall, these results suggest that the induction of miR-96 by SFA is a casual factor in the development of hepatic insulin resistance.

## Materials and Methods

### Animals and HFD-induced insulin resistance

All animal experiments were conducted in accordance with the NIH Guide for Care and Use of Laboratory Animals (1996) and were approved by the Animal Use and Care Committee at Dongguk University (approval No. IACUC-2013-007). All efforts were made to minimize animal suffering. Male C57BL/6N mice were obtained from OrientBio (Seongnam, Gyeonggi, Korea) and kept in the same temperature- and humidity-controlled holding facility on a light (12 h)-dark (12 h) cycle with food and water *ad libitum*. At 6 wks of age, the mice were fed a normal fat diet (NFD, 12% calories from fat; Purina) or a high fat diet (HFD, 60% calories from fat; Dyets Inc.) for 14 wks to establish diet-induced obese (DIO) mice. S1 Table lists the detailed compositions of the two diets. Oral glucose tolerance tests (OGTT) and insulin tolerance tests (ITT) were performed after 13 and 14 wks of feeding initiation, respectively. At the end of experiments (3 days after ITT), the mice were overnight-fasted for 12 h and sacrificed by cervical dislocation 30 min after intraperitoneal injection of the vehicle or insulin (2 U/kg of body weight; Sigma-Aldrich). The liver and gastrocnemius skeletal muscle were removed rapidly, washed three times with ice-cold PBS, and then subjected to total RNA and immunoblot analysis.

### Cell culture

HepG2, a human liver cancer cell line, was purchased from ATCC (#77400). L6 GLUT4myc myocyte was provided by Dr. Amira Klip at the Hospital for Sick Children, Toronto, Canada. HepG2 and L6 GLUT4myc cells were grown in MEMα supplemented with 10% FBS and 1% penicillin-streptomycin (Gibco) in an atmosphere containing 5% CO_2_ at 37°C. The cells from passages 3 to 10 were used for the following experiments.

### Fatty acids treatment

A bovine serum albumin (BSA)-conjugated oleate solution was purchased from Sigma-Aldrich, and a fatty acids-free BSA (Bovogen, VIC, Australia)-conjugated palmitate (Sigma-Aldrich) solution was prepared as described previously [22]. Briefly, BSA and sodium palmitate were dissolved completely in 150 mM NaCl by heating at 37°C and 70°C, respectively. The BSA solution was added slowly to the palmitate solution dropwise at 37°C with continuous stirring where the molar ratio of palmitate to BSA was 6:1. The BSA-conjugated palmitate and BSA vehicle was aliquoted and stored at -80°C. HepG2 cells were seeded at a density of 5 × 10^5^/well in a six-well plate. On the next day, the cells were treated with BSA-conjugated palmitate (0.5 mM) or oleate (0–0.5 mM) for 18 h. The control cells were treated with BSA vehicle. Where applicable, the cells were treated with or without 100 nM insulin during the last 30 min of incubation.

### Transfection of miRNA mimics and plasmids

HepG2 cells were reverse-transfected with the 200 nM mimics of miR-96, AntimiR-96 and/or scrambled control miRNA (scRNA) using G-fectin (Genolution, Seoul, Republic of Korea) according to the manufacturer’s instructions, and then seeded at a density of 3 × 10^5^/well in a six-well plate. The miRNA mimics were purchased from Genolution. For the Dual-luciferase target validation assay, L6 GLUT4myc cells were seeded at a density of 5 × 10^4^/well in a twelve-well plate. On the next day, the cells were cotransfected with 200 nM miR-96 mimic, scRNA control and/or 100 ng of plasmid containing the reporter gene using Lipofectamine 2000 (Invitrogen).

### RNA extraction, quantitative RT-PCR (*q*RT-PCR) and RT-PCR

The total cellular RNA of HepG2 cells or murine tissues (the liver and skeletal muscle) was extracted using the miRNeasy Mini Kit (Qiagen). To analyze mRNA and miRNA expression, cDNA was synthesized from the total cellular RNA (1 μg) using the miScript II RT Kit (Qiagen). The levels of miR-96, U6 snRNA or mRNA (*INSR*, *IRS-1*, *Akt2*, *GSK3β*, and *β-Actin*) expression were measured by *q*RT-PCR or RT-PCR using the miScript SYBR Green PCR Kit (Qiagen) or GoTaq Green Master Mix (Promega), respectively. The results of *q*RT-PCR were analyzed using the advanced relative quantification method in Light-Cycler 480 software (Roche Diagnostics). U6 snRNA and *β-Actin* were applied as the internal control on the expression levels of the miRNAs and mRNAs, respectively. S2 Table lists the primers (purchased from Bionics, Seoul, Republic of Korea) and PCR conditions used in this study.

### Cloning of *INSR* and *IRS-1* 3’UTRs and Dual luciferase reporter gene assay

The segment of human *INSR* 3’UTR (252 nt, INSR 3U*wt*) or *IRS-1* 3’UTR (416 nt, IRS-1 3U*wt*), containing the predicted binding site of miR-96, was amplified from the cDNA of HepG2 cells by RT-PCR using the specific primers (S2 Table). INSR 3U*wt* or IRS-1 3U*wt* was then subcloned into the pmirGLO Dual-luciferase miRNA target expression vector (Promega) using the SacI/XbaI sites. The mutated miR-96-binding sites (INSR 3U*mut* or IRS-1 3U*mut*) was prepared from the INSR 3U*wt* or IRS-1 3U*wt* using PCR-based site-directed mutagenesis with the specific primers as described in S2 Table. The Dual-luciferase target validation assay was carried out using the Dual-luciferase reporter system (Promega). Briefly, L6 GLUT4myc cells were cotransfected with miR-96, scRNA control, INSR 3U*wt*, INSR 3U*mut*, IRS-1 3U*wt*, and/or IRS-1 3U*mut* in a twelve-well plate. After 24 h transfection, the cells were lysed with the passive lysis buffer (Promega). The activities of Firefly and Renilla luciferase were determined by a Sirius L luminometer (Titertek-Berthold, Pforzheim, Germany), and the relative activity of luciferase was obtained by normalizing the ratio of Firefly/Renilla.

### Glycogen assay

HepG2 cells were reverse-transfected with miR-96 or scRNA control. After 48 h transfection, the cells were incubated in FBS-free MEMα for 4 h, and then placed in high glucose DMEM without FBS in the presence or absence of 100 nM insulin for 2 h. After washing three times with ice-cold PBS, the glycogen contents in the cells were measured using the Glycogen colorimetric assay Kit II (Biovision, CA, US) according to the manufacturer’s instructions.

### Cell lysis, immunoblotting and antibodies

HepG2 cells and the liver of mice were washed three times with ice-cold PBS and lysed using a lysis buffer (ice-cold PBS containing 1% Triton X-100, phosphatase inhibitor cocktail II (Sigma-Aldrich), and 0.2 mM PMSF) by homogenation. The lysates were mixed with 2X Laemmli buffer, and heated for 10 min at 100°C. Gel electrophoresis was carried out by SDS—PAGE on 8 or 10% resolving gels, transferred and immunoblotted with various antibodies. The anti-IRS-1 antibody was obtained from Upstate Biotechnology (Lake Placid, NY, US). The antibody against phospho-tyrosine-IRS-1 and Actin was purchased from Santa Cruz Biotechnology (Santa Cruz, CA, US). All other antibodies were purchased from Cell Signaling Technology (Danvers, MA, US). The detailed information of antibodies used in this study is listed in S3 Table. ECL Western Blotting Detection Reagent from GE Healthcare (Buckinghamshire, UK) was used for the visualization of immunoblot. The intensities of immunoblots were determined by densitometry using an Alpha Imager HP scanning system (Alpha Innotech, San Leandro, CA, US).

### *In silico* analysis and statistical analysis

The miR-96 targeting *INSR* 3’UTR and *IRS-1* 3’UTR was screened computationally using publicly available algorithms (TargetScan: www.targetscan.org, Pictar: pictar.mdc-berlin.de, and miRWalk: www.umm.uni-heidelberg.de). Data are expressed as means ± SEM. from at least three independent sets of experiments. We tested the significance of the difference using the Student's *t*-test for unpaired data.

## Results

### HFD causes metabolic dysfunction and upregulates miR-96 expression in the liver of mice

**Fig 1:**
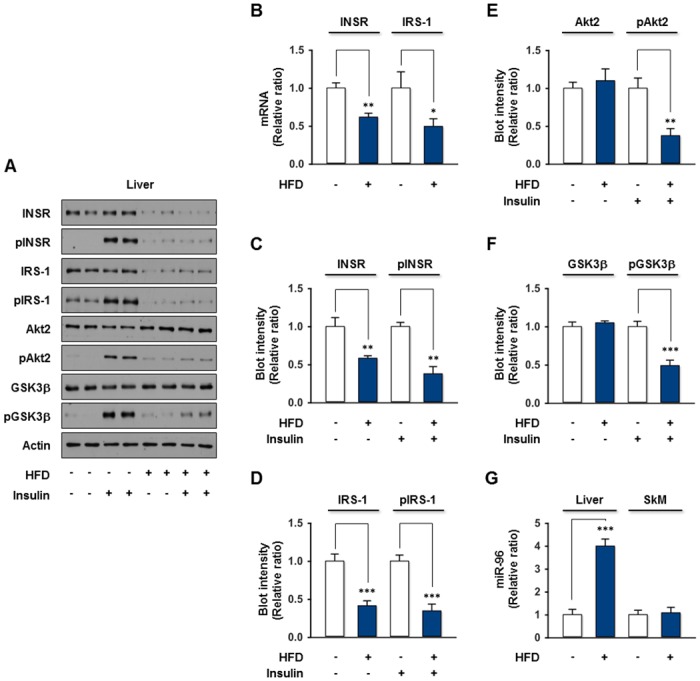
Upregulation of miR-96 by HFD in the liver of DIO mice. Mice given HFD for 14 wks exhibited hyperglycemia, impaired glucose tolerance, and impaired insulin tolerance, as shown in S1 Fig. (A) The mice were fasted overnight and sacrificed 30 min after an i.p. injection of the vehicle or insulin (2 U/kg of body weight). The representative immunoblots (2 from 7 mice/group) obtained from the liver lysates are shown. (B) The mRNA levels of *INSR* and *IRS-1* were measured by *q*RT-PCR from the liver of vehicle-injected mice. (C) The protein expression and phosphorylation of INSR was normalized to the amount of Actin. (D) The protein expression and phosphorylation of IRS-1 were normalized to the amount of Actin. (E) The protein expression of Akt2 was normalized to the amount of Actin. The level of Akt2 phosphorylation was normalized to the amount of Akt2. (F) The protein expression of GSK3β was normalized to the amount of Actin. The level of GSK3β phosphorylation was normalized to the amount of GSK3β. (G) The expression of miR-96 was measured by *q*RT-PCR from the liver and skeletal muscle (SkM). The values are expressed as the relative ratio, where the average intensity of NFD-fed mice was set to one. The values are the means ± SEM. from five individuals. **, P < 0.01; ***, P < 0.001.

Because obesity is related causally to the development of insulin resistance and the aberrant expression of miRNAs targeting the insulin signaling elements participates actively in the development of insulin resistance, HFD-induced insulin resistance was established and the miRNAs modulated by HFD were analyzed. As shown in S1 Fig, the mice fed with HFD for 14 wks showed a significant increase in the fasting blood glucose level accompanied by high body weight gain, and a reduced rate of insulin-stimulated whole body glucose disposal, as assessed by the OGTT and ITT. This result clearly suggests that HFD in mice induced hyperglycemia, impaired glucose tolerance, and impaired insulin tolerance, which are the typical characteristics of T2DM. Under these conditions, the expression of INSR and IRS-1 mRNA and protein was reduced significantly in the liver of HFD mice, whereas the expression of Akt2, GSK3β, and Actin was unaffected by HFD (Fig 1A–1F). To assess the key insulin signaling elements modulated by HFD in the liver further, the phosphorylation of INSR and its downstream signaling molecules were analyzed in the presence or absence of insulin stimulation. As expected, the phosphorylation of INSR, IRS-1, Akt2, and GSK3β induced by insulin was reduced significantly in the liver of HFD mice. These results suggest that HFD in the mice resulted in impaired hepatic insulin signaling, which was due mainly to the reduction of INSR and IRS-1 protein expression because the reduced phosphorylation level was proportional to their protein content (Fig 1A–1F).

Because the impairment of insulin signaling by HFD could be attributed most likely to the downregulation of INSR, this study assessed the alterations of *INSR* 3’UTR-targeting miRNAs in the liver of mice. We initially selected about 20 miRNAs presumably targeting *INSR* 3’UTR by TargetScan, PicTar, and miRWalk analysis for further experiments according to its targeting score to *INSR* 3’UTR, as well as quality and quantity of the results from Affymetrix miRNA array (data not shown). Interestingly, the certain miRNAs, such as miR-96, miR-140, miR-151, miR-185, miR-378, miR-455, miR-532, and miR-874, presumably targeting a 3’UTR of *INSR*, were upregulated by more than 1.5-fold in the liver of HFD mice compared to NFD-fed control, whereas the levels of other selected miRNAs remained unaffected (S2 Fig). This result is consistent with previous independent studies based on obese-type insulin resistance mice models [18, 21, 23–25]. Therefore, the upregulated miRNAs are potential candidate miRNAs for downregulating INSR expression, but none of these miRNAs has been validated to target *INSR* 3’UTR. Next, according to the confirmation of miRNAs expression by *q*RT-PCR, the level of miR-96 expression was elevated most drastically (more than 4-fold) in the liver of HFD mice but not in skeletal muscle (Fig 1G). The SFA-enriched HFD resulted in an impairment of insulin signaling via the reduction of INSR and IRS-1 protein expression with the concomitant upregulation of miR-96 in the liver of mice, and 3’UTRs of *INSR* and *IRS-1* were suggested as a potential target of miR-96 from the target prediction algorithms. Therefore, this study focused on miR-96 for further studies to evaluate its functional significance in hepatic insulin resistance.

### miR-96 is associated with an impairment of insulin signaling by palmitate

**Fig 2:**
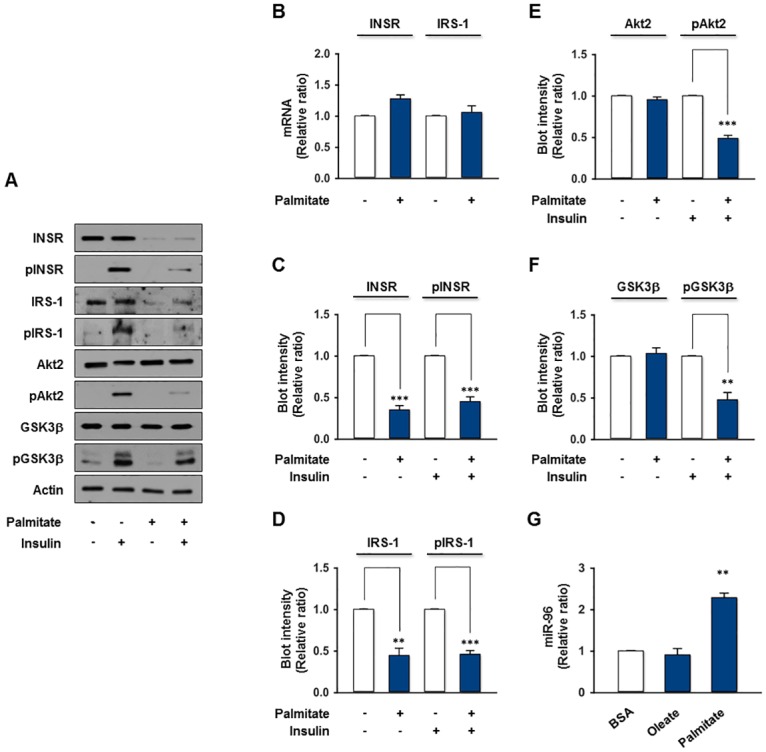
Impaired insulin signaling and upregulation of miR-96 by palmitate in HepG2 cells. HepG2 cells were treated with either the vehicle or palmitate (0.5 mM) for 18 h, and then incubated in the presence or absence of insulin (100 nM) for 30 min. (A) Representative immunoblots obtained from HepG2 cells lysates are shown. (B) The mRNA levels of *INSR* and *IRS-1* from the vehicle or palmitate-treated HepG2 cells were measured by *q*RT-PCR. (C) The protein expression or phosphorylation of INSR was normalized to the amount of Actin. (D) The protein expression or phosphorylation of IRS-1 was normalized to the amount of Actin. (E) The protein expression of Akt2 was normalized to the amount of Actin. The level of Akt2 phosphorylation was normalized to the amount of Akt2. (F) The protein expression of GSK3β was normalized to the amount of Actin. The level of GSK3β phosphorylation was normalized to the amount of GSK3β. (G) The expression of miR-96 from the vehicle BSA, oleate (0.5 mM for 18 h) or palmitate-treated HepG2 cells was measured by *q*RT-PCR. The values are expressed as the relative ratio, where the intensity of the vehicle (open column) was set to one. The values are expressed as the means ± SEM. from three independent experiments. **, P < 0.01; ***, P < 0.001.

Next, this study examined whether a high fatty acids level alone can cause impaired insulin signaling through the induction of miR-96 accompanied by a reduction of INSR in hepatocytes. Firstly, human hepatoma HepG2 cells were exposed to varying concentrations of unsaturated (oleate) fatty acid for 18 h, but oleate did not affect the expression of INSR, IRS-1, and Akt2 (S3 Fig). Therefore, HepG2 cells were treated with palmitate, which is the most abundant SFA in the diet, and the effects of palmitate on the expression and phosphorylation of insulin signaling molecules were then assessed. As shown in Fig 2, protein, but not mRNA levels of INSR and IRS-1 was reduced substantially by palmitate treatment in HepG2 cells compared to the control, while the levels of Akt2, GSK3β, and Actin expression were unaffected. In addition, palmitate led to a significant decrease in the insulin-stimulated tyrosine phosphorylation of INSR concomitantly with the reduced insulin-stimulated phosphorylation of its downstream insulin signaling molecules, such as IRS-1, Akt2, and GSK3β in HepG2 cells (Fig 2A and 2C–2F). It suggests that SFA palmitate resulted in an impairment of insulin signaling by decreasing INSR and IRS-1 expression in hepatocytes. This study then examined whether the levels of miR-96, which tentatively targets the 3’UTRs of *INSR* and *IRS-1*, were increased in the palmitate-treated HepG2 cells, because the expression of INSR and IRS-1 was decreased by palmitate. As we assumed, the level of miR-96 in HepG2 cells was upregulated drastically (approximately 2-fold) by the palmitate, but not oleate, treatment (Fig 2F), and the level of miR-96 was negatively correlated with the protein levels of INSR and IRS-1 (Fig 2A–2D), suggesting that the induction of miR-96 is closely associated with the development of insulin resistance in hepatocytes.

### *INSR* and *IRS-1* are direct targets of miR-96

**Fig 3:**
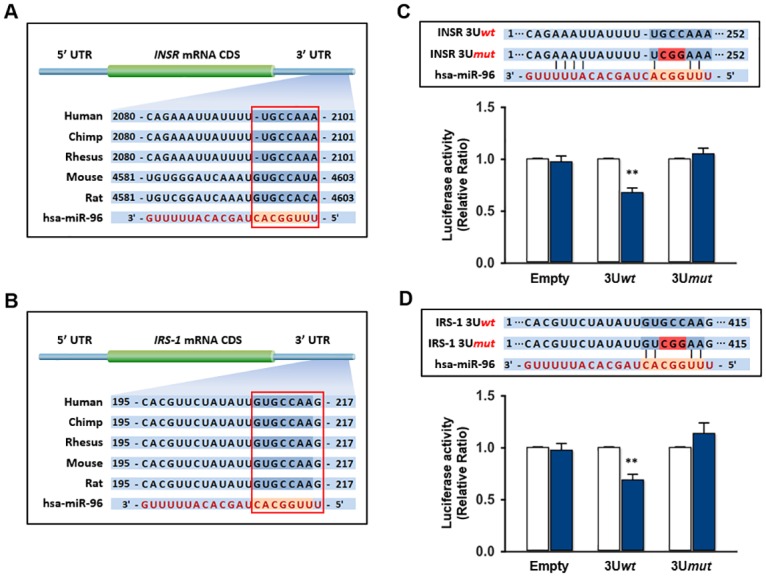
Targeting sites of miR-96 in the 3’UTRs of *INSR* and *IRS-1*, and a target validation by reporter gene assay. (A) The seed sequence (red box) of miR-96 was predicted to target *INSR* 3’UTRs. (B) The seed sequence (red box) of miR-96 was predicted to target *IRS-1* 3’UTRs. (C) The fragment of *INSR* 3’UTR was inserted downstream of a firefly luciferase open reading frame (wild-type: INSR 3U*wt*). The mutated 3’UTR of the *INSR* gene lacking the miR-96 binding site (INSR 3U*mut*) was examined (upper panel). Non-containing 3’UTR (Empty), INSR 3U*wt* or INSR 3U*mut* construct was cotransfected with either scRNA control (open column) or designated mature miR-96 mimic (closed column) into the L6 GLUT4myc cells. Luciferase activities was measured at 24 h after transfection (lower panel). (D) The fragment of *IRS-1* 3’UTR was inserted downstream of a firefly luciferase open reading frame (wild-type: IRS-1 3U*wt*). The mutated 3’UTR of the *IRS-1* gene lacking the miR-96 binding site (IRS-1 3U*mut*) was examined (upper panel). Empty, IRS-1 3U*wt* or IRS-1 3U*mut* construct was cotransfected with either scRNA control or designated miR-96 mimic into the L6 GLUT4myc cells. Luciferase activities was measured at 24 h after transfection (lower panel). The relative luciferase activities were plotted against that of each scRNA control, which was set to one. The values are expressed as the means ± SEM. from three independent experiments. **, P < 0.01.

As the upregulation of miR-96 by HFD or palmitate treatment was negatively correlated with the cellular INSR and IRS-1 level in hepatocytes (Figs 1 and 2), this study next examined whether the 3’UTRs of *INSR* and *IRS-1* are direct targets of miR-96. *In silico* target prediction analysis, such as TargetScan, PicTar, and miRWalk, showed that the 3’UTR of *INSR* or *IRS-1* mRNA contains highly conserved tentative binding sites for mature miR-96 as shown in Fig 3A and 3B. To determine if miR-96 can target *INSR* or *IRS-1* directly by interacting with its 3’UTR *in vitro*, the 3’UTR of *INSR* or *IRS-1* was cloned and inserted downstream of a luciferase reporter gene. Subsequently, L6 GLUT4myc cells were cotransfected with the mature miR-96 mimic or scRNA control using 3’UTR reporter vectors, as described in the Methods section. As indicated in Figs 1 and 2, miR-96 expression was upregulated in the hepatocytes by HFD or palmitate, and a dual luciferase-based reporter assay was conducted to determine if miR-96 regulates INSR or IRS-1 directly by binding to the 3’UTR region of *INSR* or *IRS-1*. Luciferase reporter constructs containing either a tentative miR-96 target sequence in 3’UTRs (wild-type; INSR 3U*wt* or IRS-1 3U*wt*), or nucleotides mutant of the tentative target sequence (INSR 3U*mut* or IRS-1 3U*mut*) were inserted in the pmirGLO vector (Fig 3C and 3D, upper panel). These reporter constructs were transfected transiently together with the miR-96 mimic or scRNA control. As shown in Fig 3C, cotransfection with the miR-96 mimic and reporter construct (INSR 3U*wt*) resulted in a significant reduction of luciferase activity, as compared to the scRNA control. Mutations in the tentative miR-96 binding site in *INSR* 3’UTR abrogated the suppressive effect of miR-96 (Fig 3C). This result suggests that miR-96 targets *INSR* 3’UTR directly via its binding site and then inhibits INSR expression. Similarly, the 3’UTR of the human *IRS-1* gene has been suggested to encode a tentative binding site for miR-96 (Fig 3B), and cotransfection with miR-96 and the reporter construct of *IRS-1* 3’UTR (IRS-1 3U*wt*) decreased the luciferase activity significantly, compared to the scRNA control (Fig 3D). This effect of miR-96 was abolished by mutations in its binding site (IRS-1 3U*mut*) (Fig 3D). Therefore, miR-96 participates in the repression of INSR and IRS-1 by HFD or palmitate, because INSR and IRS-1 are direct targets of miR-96.

### INSR and IRS-1 are suppressed by miR-96 at the post-transcriptional level

**Fig 4:**
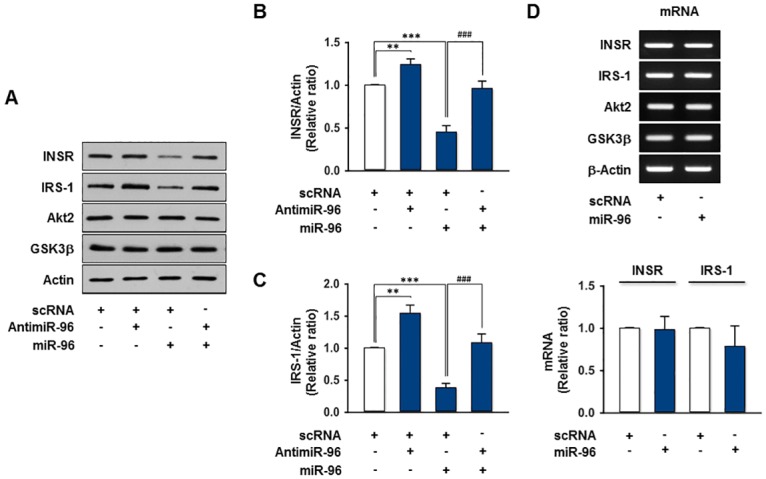
Effect of miR-96 on the INSR and IRS-1 expression. (A-C) HepG2 cells were reverse-transfected with scRNA (100 nM), AntimiR-96 (100 nM), and/or miR-96 (100 nM) mimic. After 48 h transfection, the expression of insulin signaling intermediates was analyzed by immunoblotting. Representative immunoblots obtained from HepG2 cells lysates were shown in A. (B) The expression of INSR was normalized to the amount of Actin. (C) The expression of IRS-1 was normalized to the amount of Actin. (D) HepG2 cells were reverse-transfected with 200 nM of miR-96 mimic or scRNA control. The mRNA levels were analyzed at 24 h after reverse-transfection by RT-PCR (upper panel) and *q*RT-PCR (lower panel). Values are means ± SEM. **, P < 0.01; ***, P < 0.001 vs. scRNA control; ###, p < 0.001 scRNA + miR-96 vs. AntimiR-96 + miR-96.

As miR-96 interacts authentically with *INSR* and *IRS-1* mRNAs by binding to their 3’UTR regions, the induction of miR-96 should repress INSR and IRS-1 expression, leading to an impairment of insulin signaling in hepatocytes. To examine this further, the miR-96 mimic was transfected into the HepG2 cells by reverse-transfection and the expression levels of INSR, IRS-1 and insulin signaling intermediates were assessed. To measure the transfection efficiency of the miR-96 mimics and scRNA control, the level of miR-96 expression was analyzed by *q*RT-PCR in HepG2 cells before and after transfection. Based on an *q*RT-PCR analysis, the level of miR-96 in HepG2 cells was increased > 1,000-fold by transfection with 200 nM of the miR-96 mimics, compared to endogenous miR-96 (data not shown). Therefore, 200 nM of the miR-96 mimic and AntimiR-96, a 2’-O-methyl-modified antisense oligonucleotide against mature miR-96, was adopted for reverse-transfection. The overexpression of miR-96 in HepG2 cells exhibited a significant decrease in the levels of INSR and IRS-1 protein expression compared to the scRNA control, whereas the protein expression of the insulin signaling molecules, such as Akt2 and GSK3β, were unaffected (Fig 4A–4C). Moreover, transfection with AntimiR-96 revoked the repression of INSR and IRS-1 by miR-96 almost completely, whereas the levels of INSR and IRS-1 were increased by the inhibition of endogenous miR-96 using AntimiR-96 (Fig 4A–4C). On the other hand, there were no significant changes in the mRNA levels of *INSR* and the insulin signaling molecules, such as *IRS-1*, *Akt2*, and *GSK3β*, according to RT-PCR and *q*RT-PCR (Fig 4D), indicating that miR-96 suppresses INSR and IRS-1 at the post-transcriptional level. Overall, miR-96 promotes the downregulation of INSR and IRS-1 in HepG2 cells.

### Overexpression of miR-96 inhibits insulin signaling and glycogen synthesis

**Fig 5:**
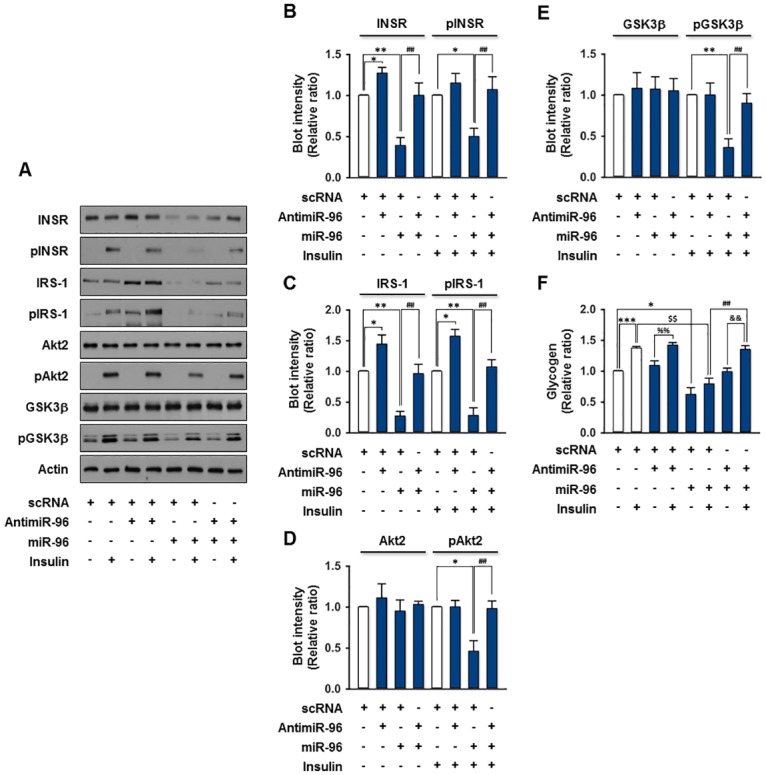
Impairment of insulin signaling and glycogen synthesis by miR-96. (A-E) HepG2 cells were reverse-transfected with the scRNA (100 nM), AntimiR-96 (100 nM) and/or miR-96 (100 nM) mimic. After 48 h transfection, the cells were incubated in the presence or absence of insulin (100 nM) for 30 min and subjected to immunoblotting. (A) Representative immunoblots obtained from HepG2 cells lysates are shown. (B) The expression and phosphorylation of INSR were normalized to the amount of Actin. (C) The expression and phosphorylation of IRS-1 were normalized to the amount of Actin. (D) The protein expression of Akt2 was normalized to the amount of Actin. The level of Akt2 phosphorylation was normalized to the amount of Akt2. (E) The protein expression of GSK3β was normalized to the amount of Actin. The level of GSK3β phosphorylation was normalized to the amount of GSK3β. (E) The glycogen contents were quantified in the presence or absence of insulin (100 nM) from the scRNA control, AntimiR-96 and/or miR-96 mimic-transfected HepG2 cells. The values are expressed as the relative ratio, where the intensity of the scRNA control was set to one. The values are expressed as the means ± SEM. from three independent experiments. *, P < 0.05; **, P < 0.01; ***, P < 0.001 vs. scRNA control; $$, P < 0.01 scRNA + insulin vs. miR-96 + insulin; %%, P < 0.01 AntimiR-96 vs. AntimiR-96 + insulin; &&, P < 0.01 AntimiR-96 + miR-96 vs. AntimiR-96 + miR+96 + insulin; ##, P < 0.01 miR-96 vs. AntimiR-96 + miR-96.

As INSR and IRS-1 expression is suppressed at the post-transcriptional level by miR-96 in hepatocytes, this study further examined whether the upregulation of miR-96 causes insulin resistance in hepatocytes. The overexpression of miR-96 alone in HepG2 cells decreased the insulin-stimulated phosphorylation of INSR significantly at the receptor level of insulin signaling, with the concomitant repression of INSR (Fig 5A and 5B). Moreover, the phosphorylation of the downstream signaling molecules of INSR, such as IRS-1, Akt2, and GSK3β, was also reduced dramatically by the overexpression of miR-96 alone (Fig 5A–5E). This effect is attributed mainly to the reduced expression of INSR. Although, on the other hand, overexpression of AntimiR-96 alone increased slightly the expression of INSR and IRS-1, it is only seemed to increase insulin-stimulated phosphorylation of IRS-1 significantly (Fig 5A–5C). However, cotransfection of AntimiR-96 with miR-96 almost completely rescued the inhibition of expression and insulin-stimulated phosphorylation of INSR and IRS-1 caused by miR-96 (Fig 5A–5C). Finally, the modulation of glycogen synthesis by miR-96 was assessed in the presence or absence of insulin and/or AntimiR-96 in hepatocytes (Fig 5F). In the control, insulin increased significantly the level of GSK3β phosphorylation and glycogen synthesis, whereas insulin-stimulated phosphorylation of GSK3β and glycogen synthesis were abolished almost completely by the overexpression of miR-96 alone (Fig 5E and 5F). Furthermore, the cotransfection of Antimir-96 with miR-96 recovered impairment of insulin-stimulated glycogen synthesis induced by miR-96 overexpression, indicating that miR-96 regulates the glycogen synthesis in hepatocytes (Fig 5F). Therefore, the upregulation of miR-96 provokes an impairment of insulin signaling and glycogen synthesis in hepatocytes through the repression of INSR and IRS-1, and that miR-96 is a causal factor for SFA-induced hepatic insulin resistance.

## Discussion

Obesity is closely associated with the dysregulation of glucose utilization and cellular metabolism, as well as cell survival in many cells [2, 5]. In particular, the excessive intake of SFA increases the accumulation of DAG, mitochondrial dysfunction, inflammation, and oxidative stress in the liver, eventually provoking insulin resistance, T2DM, and metabolic syndrome [7, 8]. Over the last two decades, accumulating evidence has suggested that certain miRNAs targeting the molecules in the insulin signaling cascade are expressed aberrantly in SFA-induced obesity, leading to insulin resistance and T2DM [14]. The present study revealed for the first time that (i) the expression of miR-96 is upregulated in the liver of HFD mice, as well as in the palmitate-treated HepG2 cells; (ii) miR-96 targets *INSR* and *IRS-1* 3’UTRs directly and downregulates INSR and IRS-1 expressions at the post-transcriptional level in hepatocytes; and (iii) the overexpression of miR-96 impairs hepatic insulin signaling and the glycogen metabolism through the repression of INSR and IRS-1. Therefore, these results reveal a novel mechanism whereby the induction of miR-96 plays a crucial role in the development of hepatic insulin resistance in SFA-induced obesity.

Hsa-miR-96 located on chromosome 7q32.2 is a member of the miR-183 family, which consists of three highly conserved miRNAs (miR-96, miR-182 and miR-183) in vertebrates [26]. Although the miR-183 family exhibits the conservation of their genomic organization and sequence homology, many lines of evidence indicate that the levels of these miRNAs can be regulated individually by transcription, DNA methylation, and post-transcriptional processing [26, 27]. Based on recent findings, the members of the miR-183 family, despite their sequences being dissimilar, are involved in a wide variety of normal physiological processes and pathological conditions, such as cell proliferation, apoptosis, immunity, and metabolism [26, 28, 29]. Among them, the role and biological significance of miR-96 have focused mainly on the oncogenesis and cancer progression in various malignancies, such as the liver, stomach, and breast cancer [29–31]. Therefore, miR-96 is recognized as an oncomiR that facilitates the development of malignancies by promoting the growth, proliferation, and survival of cancer cells [32, 33]. On the other hand, the metabolic influence of miR-96 was first reported in pancreatic β-cells [34]. Insulin secretion from pancreatic β-cells was inhibited substantially by miR-96, which increased the levels of the granuphilin/Slp4 protein, a potent inhibitor of insulin exocytosis, and decreased the expression of Noc2, a Rab27A-binding protein [34]. Recently, miR-96 was reported to be upregulated by the depletion of mitochondrial DNA and targeted IRS-1 directly in SK-Hep1 cells [35], suggesting that the role of miR-96 in insulin resistance results from a mitochondrial dysfunction. Because obesity is an established risk factor for oncogenesis, metastasis, chemoresistance, and a poorer prognosis [36], even though the biological mechanisms underlying the relationship between obesity and cancer are complex and not well understood [37], the current study showed that obesity-induced miR-96 regulates insulin signaling, which provides novel insights into the molecular basis underlying the obesity-driven pathogenesis of insulin resistance, β-cell failure, and cancer progression. Hence, miR-96 is proposed as a key player in the crosstalk between metabolic diseases and cancer, as well as the detrimental factors for obesity-induced lipotoxicity and insulin resistance. Therefore, the clinically applicable specific inhibitors for miR-96 may have developed as potential tools for therapeutic applications in the metabolism and cancer, even though further study in this subject is clearly warranted.

In this study, the expression of miR-96 was increased significantly in the liver of HFD mice and palmitate-treated hepatocytes. Although only a limited number of reports regarding the differential expression of miR-96 are available, recent studies based on an analysis of various metabolic dysfunctions provided not only supporting data for these results, but also showed the potential significance of miR-96 in hepatic insulin sensitivity. Members of the miR-183 family were upregulated in the liver of NAFLD patients [38] and obese diabetic animal models, such as *db/db* and *ob/ob* mice [39, 40]. Moreover, an increase in miR-96 was also observed in the retinas of diabetic retinopathy rats [41]. Interestingly, miR-96 was upregulated in the liver of insulin resistant mice injected with resistin [42], which is considered a potential adipokine responsible for obesity-mediated insulin resistance, T2DM, and inflammation [43]. In addition, the expression of resistin was higher in the plasma of HFD-fed mice and its inhibition improved HFD-induced hepatic insulin resistance [44]. Although there is no evidence of a direct molecular mechanism involved in the transcriptional activation of miR-96 expression by obesity and resistin, an increased miR-96 level could contribute to the development of insulin resistance induced by resistin or obesity.

Although the direct molecular mechanisms demonstrating the transcriptional regulation of miR-96 expression have never been reported, the upregulation of miR-96 in hepatocytes might be triggered by the palmitate or HFD-induced activation of certain transcription factors. *In silico* analysis of the transcription factor binding sites suggests that several tentative regulators activated in obesity or HFD may bind to the specific regions located on the promoter of miR-96. Among them, SREBPs, which play key roles in the biosynthesis of cholesterol and fatty acids, are activated by HFD and their activation is linked causally to diabetic fatty liver and carbohydrate-induced hyperlipidemia [45]. Recently, Jeon, et al. reported that the promoter for miR-96 on mouse chromosome 6 is a direct target of SREBP activation [46]. Accordingly, the level of miR-96 expression is most likely to be induced by SREBPs activation, resulting from palmitate or HFD. Moreover, miR-96 can provoke a rapid increase in nuclear SREBPs and lipid synthesis in the liver of mice via the negative regulation of INSIG-2, which is a key molecule for retaining the SREBP-precursor in the ER [46]. Therefore, the activation of SREBPs in the liver by SFA may be a substantial regulatory mechanism for miR-96 induction. In addition, similar to SREBPs, PPARγ are responsible for lipid accumulation in the liver and the expression of several genes important for the lipid metabolism [47]. According to global microarray analysis, the expression of miR-96 was increased by PPARγ activation in differentiating 3T3-L1 adipocytes [48]. Moreover, PPARγ is colocalized with an adipogenic transcription factor, C/EBPα [49], which is also activated by HFD-fed mice [50]. In this regard, the activation of PPARγ co-operated with C/EBPα in obesity also play an important regulatory role for the upregulation of miR-96 in the liver. It is also noteworthy that several lines of evidence imply the induction of miR-96 through an XBP1-dependent mechanism under ER stress induced by HFD. XBP1 is known to bind to the promoter region of C/EBPα, which promotes adipogenesis and lipid [51], and is activated ER stress conditions, such as HFD and obesity [52]. Although further study is warranted, these studies suggest that miR-96 may be a novel culprit in the vicious cycle between the hepatic lipid accumulation and insulin resistance. In conclusion, the expression of miR-96 is upregulated substantially in the liver of HFD mice as well as hepatocytes treated with SFA palmitate. miR-96 suppresses INSR and IRS-1 by targeting their 3’UTRs directly. The overexpression of miR-96 results in the drastic repression of INSR and IRS-1 protein expression, which subsequently leads to an impairment of insulin signaling and glycogen synthesis in hepatocytes. Overall, these results show that miR-96 induced by SFA is involved in the development of hepatic insulin resistance, which may in turn lead to T2DM.

## Supporting Information

S1 Fig HFD induces obesity, hyperglycemia, impaired glucose tolerance, and impaired insulin tolerance in C57BL/6N male mice. (A) The mice were fed either NFD or HFD for 14 weeks and the body weights were measured every week. The body weights increased significantly after 2 weeks of HFD-feeding. (B) The mice showed an increased fasting blood glucose after 14 weeks of HFD-feeding, indicating that 14 weeks of the HFD led to hyperglycemia in mice. (C) The NFD-fed and HFD-fed mice underwent an oral glucose tolerance test (OGTT). The mice were fasted overnight and administrated a 20% glucose solution orally (10ml/kg of body weight) using an 18-gauge gavage needle. The plasma glucose level in the tail of the mice were measured every 30 min using a Blood Glucose Monitoring System (SD Biosensor, Seoul, Republic of Korea). (D) OGTT was analyzed by calculating the area under curve (AUC). (E) The NFD-fed and HFD-fed mice underwent an insulin tolerance test (ITT). The mice were fasted for 3 h and i.p. injected insulin (1U/kg of body weight). The plasma glucose level in the tail of the mice was measured at 15, 30, 60, 90, and 120 min using a Blood Glucose Monitoring System. (F) ITT was analyzed by calculating the AUC. The values are expressed as the mean ± SEM. from five mice, for the NFD control (circle or open column) and HFD (circle or closed column). *, P < 0.05; **, P < 0.01; ***, P < 0.001 vs. NFD control.
(PDF)

S2 Fig Expression of miRNAs presumably targeting *INSR* 3’UTR in the liver of HFD-fed mice. The expression levels of miRNAs that predicted to target *INSR* 3’UTR were analyzed using Affymetrix Genechip miRNA 4.0. The labeled RNA was quantified, fractionated and hybridized to the miRNA microarray according to the standard procedures provided by the manufacture. The chips were stained and scanned using a Genechip Fluidics Station 450 (Affymetrix, Santa Clara, California, United States) and Affymetrix GCS 3000 scanner (Affymetrix). The signal values were computed using the Affymetrix^®^ GeneChip^™^ Command Console software, and are expressed as the relative ratio, where the value of the NFD-fed control was set to one. The blue columns represent the upregulated miRNAs using a fold change cutoff of 1.5 or greater.
(PDF)

S3 Fig Expression of INSR and IRS-1 by oleate in HepG2 cells. HepG2 cells were treated either with the vehicle or oleate (0.1–0.5 mM) for 18 h. (A) Representative immunoblots obtained from HepG2 cells lysates are shown. (B) The protein expression of INSR was normalized to the amount of Actin. (C) The protein expression of IRS-1 was normalized to the amount of Actin. The values are expressed as the relative ratio, where the intensity of the vehicle (open column) was set to one. The values are expressed as the means ± SEM. from three independent experiments.
(PDF)

S1 Table Diet composition of NFD and HFD. (PDF)

S2 Table Primer lists and PCR conditions for *q*RT-PCR, RT-PCR and cloning. (PDF)

S3 Table Antibodies used in this study. (PDF)

## Abbreviations

Akt2: v-akt murine thymoma viral oncogene homolog 2
C/EBPα: CCAAT/enhancer binding protein alpha
DAG: diacylglycerol
ER: endoplasmic reticulum
GSK3β: glycogen synthase kinase 3 beta
Hsa: human
IKK: I kappa B kinase
JNK: c-Jun N-terminal kinase
PDK-1: phosphoinositide-dependent kinase-1
PI3K: phosphoinositide-3-kinase
PKC: protein kinase C
PPARγ: peroxisome proliferator activated receptor gamma
SREBP: sterol regulatory element binding protein
XBP1: X-box binding protein 1
