# Supplementary figures and images for: Induction of miR-96 by Dietary Saturated Fatty Acids Exacerbates Hepatic Insulin Resistance through the Suppression of INSR and IRS-1
Source: PLoS One. 2016 Dec 30;11(12):e0169039. doi: 10.1371/journal.pone.0169039 (PMC5201257; doi:10.1371/journal.pone.0169039)

**S1 Fig.**

**A**

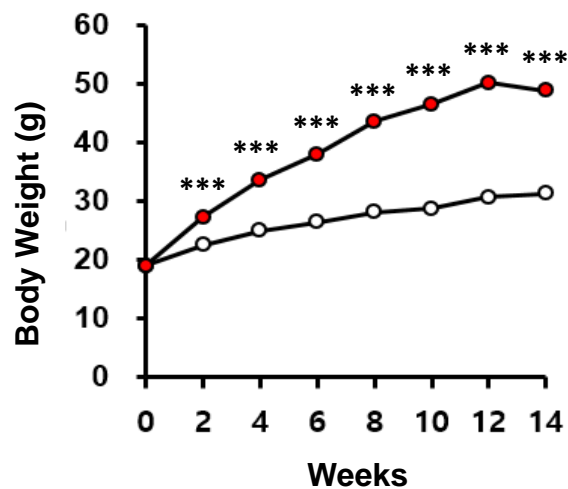

**C**

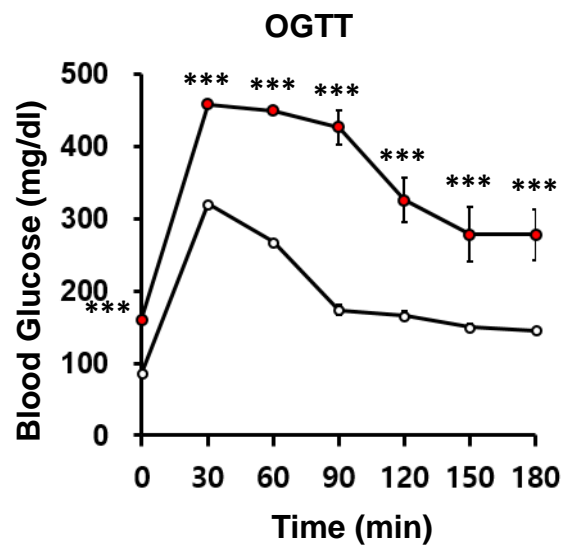

**E**

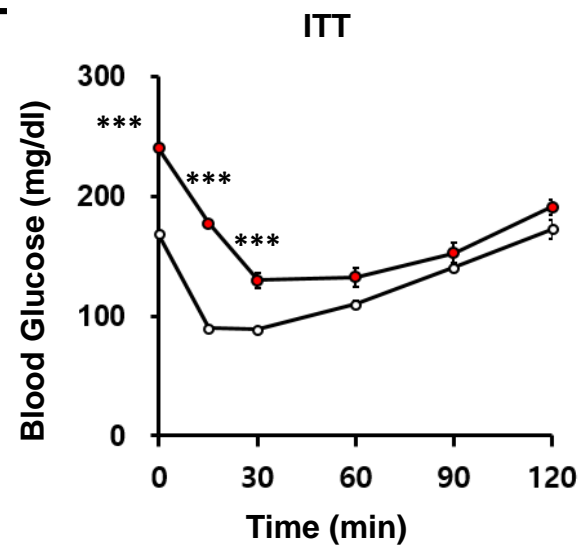

**B**

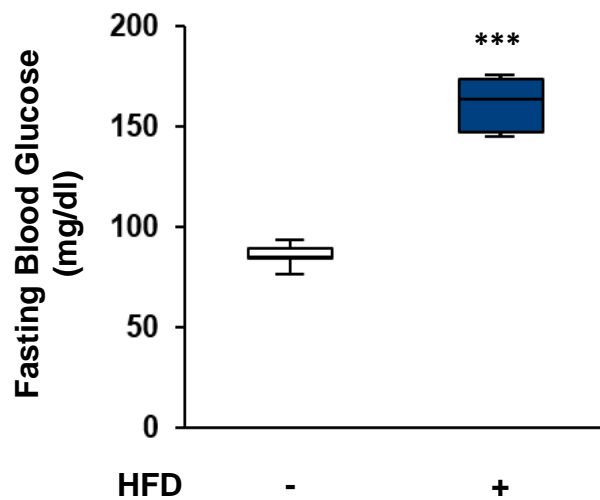

**D**

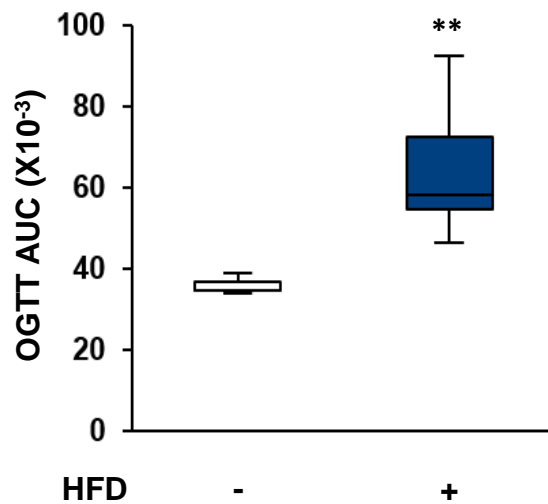

**F**

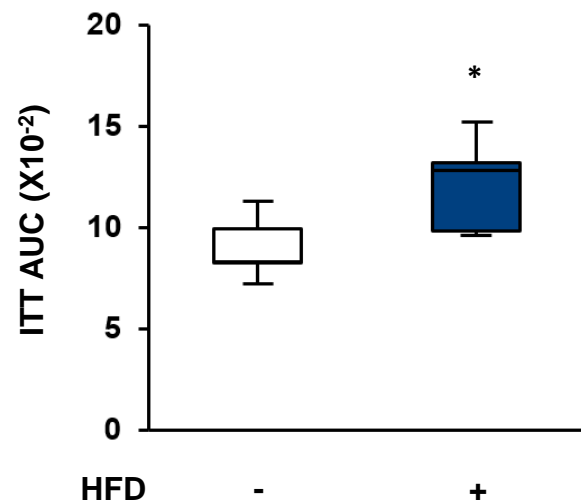

Supplement: S1 Fig — (A) The mice were fed either NFD or HFD for 14 weeks and the body weights were measured every week. The body weights increased significantly after 2 weeks of HFD-feeding. (B) The mice showed an increased fasting blood glucose after 14 weeks of HFD-feeding, indicating that 14 weeks of the HFD led to hyperglycemia in mice. (C) The NFD-fed and HFD-fed mice underwent an oral glucose tolerance test (OGTT). The mice were fasted overnight and administrated a 20% glucose solution orally (10ml/kg of body weight) using an 18-gauge gavage needle. The plasma glucose level in the tail of the mice were measured every 30 min using a Blood Glucose Monitoring System (SD Biosensor, Seoul, Republic of Korea). (D) OGTT was analyzed by calculating the area under curve (AUC). (E) The NFD-fed and HFD-fed mice underwent an insulin tolerance test (ITT). The mice were fasted for 3 h and i.p. injected insulin (1U/kg of body weight). The plasma glucose level in the tail of the mice was measured at 15, 30, 60, 90, and 120 min using a Blood Glucose Monitoring System. (F) ITT was analyzed by calculating the AUC. The values are expressed as the mean ± SEM. from five mice, for the NFD control (circle or open column) and HFD (circle or closed column). *, P < 0.05; **, P < 0.01; ***, P < 0.001 vs. NFD control. (PDF) [file pone.0169039.s001.pdf]

S2 Fig.

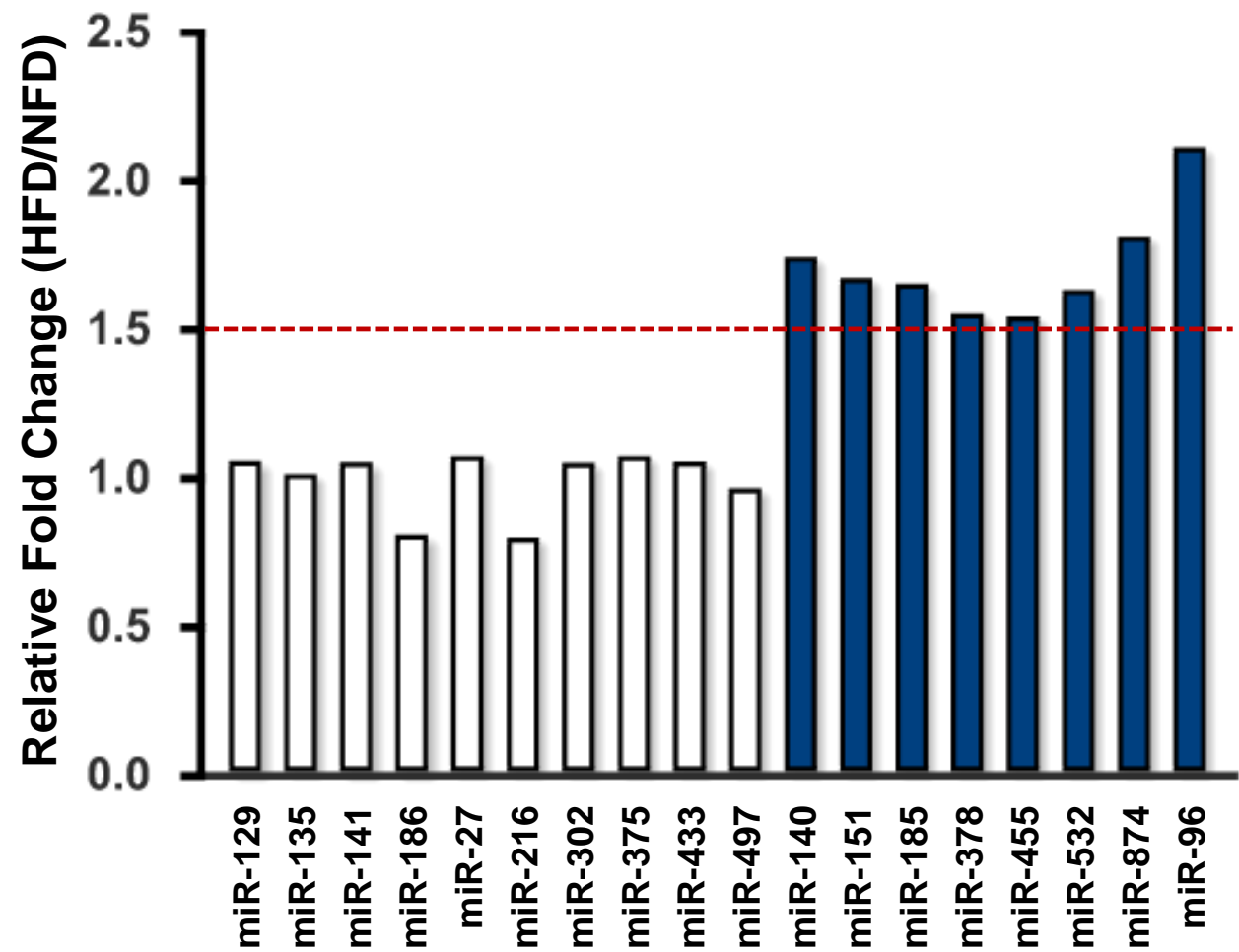

Supplement: S2 Fig — The expression levels of miRNAs that predicted to target INSR 3’UTR were analyzed using Affymetrix Genechip miRNA 4.0. The labeled RNA was quantified, fractionated and hybridized to the miRNA microarray according to the standard procedures provided by the manufacture. The chips were stained and scanned using a Genechip Fluidics Station 450 (Affymetrix, Santa Clara, California, United States) and Affymetrix GCS 3000 scanner (Affymetrix). The signal values were computed using the Affymetrix® GeneChip™ Command Console software, and are expressed as the relative ratio, where the value of the NFD-fed control was set to one. The blue columns represent the upregulated miRNAs using a fold change cutoff of 1.5 or greater. (PDF) [file pone.0169039.s002.pdf]

S3 Fig.

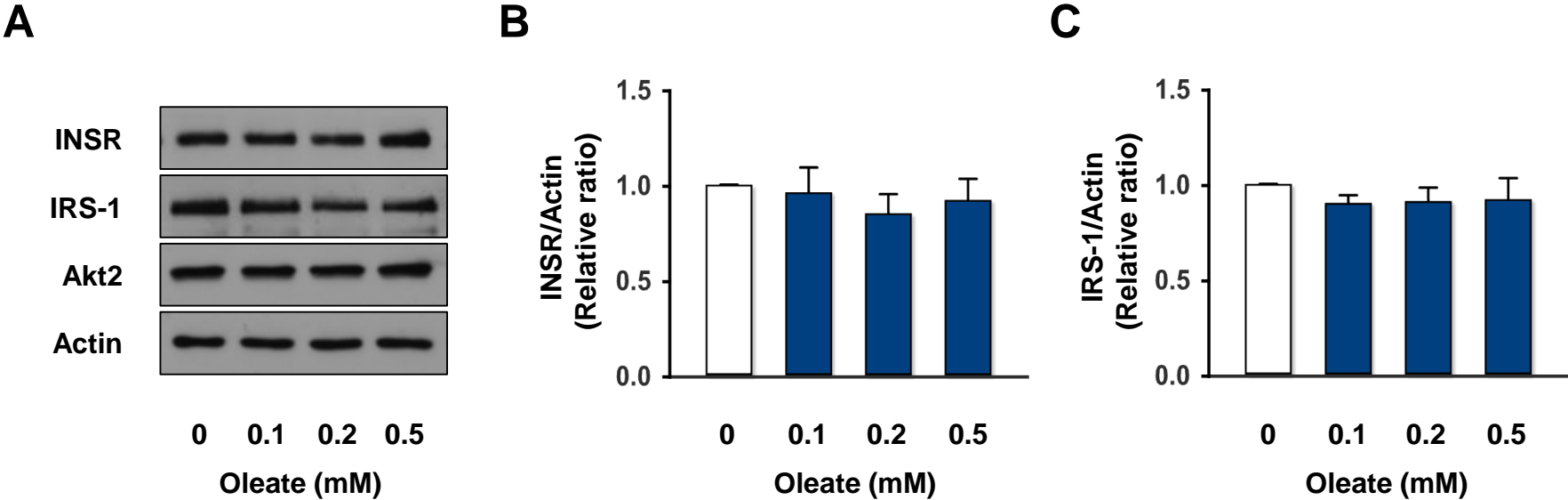

Supplement: S3 Fig — HepG2 cells were treated either with the vehicle or oleate (0.1–0.5 mM) for 18 h. (A) Representative immunoblots obtained from HepG2 cells lysates are shown. (B) The protein expression of INSR was normalized to the amount of Actin. (C) The protein expression of IRS-1 was normalized to the amount of Actin. The values are expressed as the relative ratio, where the intensity of the vehicle (open column) was set to one. The values are expressed as the means ± SEM. from three independent experiments. (PDF) [file pone.0169039.s003.pdf]
