# Supplementary material for: Induction of miR-96 by Dietary Saturated Fatty Acids Exacerbates Hepatic Insulin Resistance through the Suppression of INSR and IRS-1
Source: PLoS One. 2016 Dec 30;11(12):e0169039. doi: 10.1371/journal.pone.0169039 (PMC5201257; doi:10.1371/journal.pone.0169039)
Supplement: S1 Table — (PDF) [file pone.0169039.s004.pdf]

S1 Table. Diet composition of NFD and HFD

(A) Normal fat diet (NFD; Purina Laboratory Rodent Diet 38057)

Calories (%)		Fat component of total fat (gram%)		
		Saturated fatty acid	Unsaturated Fatty acids	Unidentified fat
Fat	12.41%	Arachidonic Acid (4.42%)	Omega-3 fatty acid (24.55%)<br>Linoleic acid (24.15%)<br>Linolenic acid (2.65%)	Unidentified (44.23%)
Carbohydrate	63.07%			
Protein	24.52%			
Total	100%	4.42% of total fat	51.35% of total fat	44.23% of total fat

(B) High fat diet (HFD; D12492)

Calories (%)		Fat component of total fat (gram%)	
		Saturated fatty acid	Unsaturated Fatty acids
Fat	60%	Palmitic acid (19.64)<br>Stearic acid (10.59%)<br>Myristoleic acid (1.10%)<br>Lau acid (0.8%)<br>Margaric acid (0.35%)<br>Arachidic acid (0.16%)<br>Pentadecanoic acid (0.08%)<br>Capric acid (0.04%)	Oleic acid (33.68%)<br>Linoleic acid (28.37%)<br>Linolenic acid (2.04%)<br>Arachidonic acid (1.78%)<br>Palmitoleic acid (1.33%)<br>Docosapentaenoic acid (0.78%)
Carbohydrate	20%		
Protein	20%		
Total	100%	32% of total fat	68% of total fat
