# Supplementary material for: Induction of miR-96 by Dietary Saturated Fatty Acids Exacerbates Hepatic Insulin Resistance through the Suppression of INSR and IRS-1
Source: PLoS One. 2016 Dec 30;11(12):e0169039. doi: 10.1371/journal.pone.0169039 (PMC5201257; doi:10.1371/journal.pone.0169039)
Supplement: S2 Table — (PDF) [file pone.0169039.s005.pdf]

S2 Table. Primer lists and PCR conditions for *q* RT-PCR, RT-PCR and cloning

(A) Human primer lists for *q* RT-PCR

Gene	Primer sequence (5´-3´)		Product size	Annealing Temperature	Concentration		Cycle
					cDNA	Primer	
miR-96	F.P	GACTCTGTTTGGCACTAGCACAT	87	55	2 ng/μl	0.5 μM	40
miRNA universal primer	R.P	miScript universal primer (Qiagen)					
U6	F.P	CTCGCTTCGGCAGCACA	94				
	R.P	AACGCTTCACGAATTTGCGT					
INSR	F.P	GCTGGTGTCTGAGCTTCAG	406	58			
	R.P	CTCGCACCCTTGAGAAGAACC					
IRS1	F.P	GTTTCCAGAAGCAGCCAGAG	483	60			
	R.P	ACTCTCTCCACCCAACGTGA					
β-Actin	F.P	TCACCCACACTGTGCCCATCTACGA	348	58			
	R.P	GGATGCCACAGGATTCCATACCCA					

(B) Mouse primer lists for *q* RT-PCR

Gene	Primer sequence (5´-3´)		Product size	Annealing Temperature	Concentration		Cycle
					cDNA	Primer	
miR-96	F.P	GACTCTGTTTGGCACTAGCACAT	87	55	2 ng/μl	0.5 μM	40
miRNA universal primer	R.P	miScript universal primer (Qiagen)					
U6	F.P	CTCGCTTCGGCAGCACA	94				
	R.P	AACGCTTCACGAATTTGCGT					
INSR	F.P	AGGCTCCCGTCTCTTCTTCAA	226	60			
	R.P	GACATCCCCACATTCCTCGTT					
IRS1	F.P	GTTTCCAGAAGCAGCCAGAG	474	60			
	R.P	ACTCTCTCCACCCAACGTGA					
β-Actin	F.P	TCACCCACACTGTGCCCATCTACGA	348	58			
	R.P	GGATGCCACAGGATTCCATACCCA					

(C) Human primer lists for RT-PCR

Gene	Primer sequence (5´-3´)		Product size	Annealing Temperature	Concentration		Cycle
					cDNA	Primer	
INSR	F.P	GCTGGTGTCTGAGCTTCAG	406	58	2 ng/μl	0.5 μM	30
	R.P	CTCGCACCCTTGAGAAGAACC					
IRS1	F.P	GTTTCCAGAAGCAGCCAGAG	483	60			
	R.P	ACTCTCTCCACCCAACGTGA					
Akt2	F.P	GAGGTCATGGAGCACAGGTT	195	58			
	R.P	CTGGTCCAGCTCCAGTAAGC					
GSK3β	F.P	ATTACGGGACCCAAATGTCA	217				
	R.P	TGCAGAAGCAGCATTATTGG					
β-Actin	F.P	TCACCCACACTGTGCCCATCTACGA	348				
	R.P	GGATGCCACAGGATTCCATACCCA					

(D) Primer lists for 3'UTR wild-type cloning

Gene	Primer sequence (5´-3´)		Product size	Annealing Temperature	Concentration		Cycle
					cDNA	Primer	
INSR 3Uwt	F.P	AAAAAGAGCTCCTTAAGAATGGGATAGAGTC	252	58	2 ng/μl	0.5 μM	35
	R.P	AAAAATCTAGATAGGTACAGACCCTCATAT					
IRS1 3Uwt	F.P	AAAAAGAGCTCTCAGTAGCTCAACTGGACAT	437				
	R.P	AAAAATCTAGAACAATGATGCTTTGTCGTAC					

(E) Primer lists for 3'UTR mutant cloning

Gene	Primer sequence (5´-3´)		Product size	Annealing Temperature	Concentration		Cycle
					wt plasmid	Primer	
INSR 3Umut	F.P	GACCAATAGCTGGACCTTTCATATTTTGG	62	58	2 ng/μl	0.5 μM	35
	R.P	CCAAAATATGAAAGGTCCAGCTATTGGTC	226				
IRS-1 3Umut	F.P	TTCTATATTGTCGGAAGCGA	237				
	R.P	TCGCTTCCGACAATATAGAA	221				
