# Supplementary material for: Induction of miR-96 by Dietary Saturated Fatty Acids Exacerbates Hepatic Insulin Resistance through the Suppression of INSR and IRS-1
Source: PLoS One. 2016 Dec 30;11(12):e0169039. doi: 10.1371/journal.pone.0169039 (PMC5201257; doi:10.1371/journal.pone.0169039)
Supplement: S3 Table — (PDF) [file pone.0169039.s006.pdf]

S3 Table. Antibodies used in this study

Antibody	Company	Cat. No.	Host	Dilution ratio
INSR	Cell Signaling Technology	#3020	Mouse	1:3,000
phospho-INSR (Tyr 1150/1151)	Cell Signaling Technology	#3024	Rabbit	1:3,000
IRS-1	Merck Millipore	#06-248	Rabbit	1:5,000
phospho-IRS-1 (Tyr 632)	Santa Cruz Biotechnology	sc-17196-R	Rabbit	1:2,000
Akt	Cell Signaling Technology	#9272	Rabbit	1:10,000
phospho-Akt2 (Ser 474)	Cell Signaling Technology	#8599	Rabbit	1:10,000
GSK3β	Cell Signaling Technology	#9315	Rabbit	1:10,000
phospho-GSK3α/β (Ser 21/9)	Cell Signaling Technology	#9331	Rabbit	1:10,000
Actin	Santa Cruz Biotechnology	sc-1616-R	Rabbit	1:10,000
Anti-rabbit HRP	Santa Cruz Biotechnology	sc-2004	Goat	1:10,000
Anti-mouse HRP	PIERCE	#1858413	Goat	1:3,000
